# Neurodevelopmental Conditions Amongst Care-Experienced Children: A Population-Based Cohort Study Using Linked Health, Social Care, and Education Records from Wales

**DOI:** 10.23889/ijpds.v11i1.3798

**Published:** 2026-10-06

**Authors:** Helen Hodges, Grace A Bailey, Laura E Cowley, Sarah Thompson, Lucy J Griffiths

**Affiliations:** 1 Population Data Science, Swansea University Medical School, Swansea University, Swansea, SA2 8PP; 2 Children’s Social Care Research and Development Centre (CASCADE), Social Science Research Institute, Cardiff University, Cardiff, CF24 4HQ; 3 Joint first authorship; 4 Posthumous authorship

**Keywords:** Children’s social care, child welfare, children looked after, neurodivergence, neurodiverse, administrative data, data linkage, neurodevelopmental disorders, neurodevelopmental conditions

## Abstract

**Background:**

Care-experienced children are a vulnerable group with higher exposure to inequalities. Nationwide evidence on the proportion of children with neurodevelopmental conditions (NDCs) and age at diagnosis among those with care experience is limited.

**Methods:**

We conducted a retrospective population-based cohort study using linked administrative data from the Secure Anonymised Information Linkage Databank in Wales. NDCs were identified using healthcare and education records among a cohort of children born between 2002 and 2006, followed to age 16. Proportions of NDCs, median age at first recorded NDC and the source of identification were compared between children with and without care experience using chi-square tests and Mann-Whitney U tests.

**Results:**

The study included 126,980 children, comprising 123,830 children without care experience, and 3,150 with care experience. 30% of children had a recorded NDC. Proportions were higher among those with care experience compared to those without (5,606 vs 2,888 per 10,000), particularly for learning difficulties, autism spectrum disorder (ASD), and attention deficit hyperactivity disorder (ADHD). Among children with NDCs, median age at recorded diagnosis was younger for those with care experience, particularly ASD (7.4 vs 10.3 years). Source of recorded diagnosis differed by NDC and care experience. For ASD, diagnosis was similarly distributed across health and education among those without care experience, whereas among care-experienced children, more were diagnosed through healthcare. Learning difficulties were predominantly diagnosed in educational settings, while ADHD was predominantly identified through healthcare.

**Conclusion:**

Care-experienced children have higher recorded proportions of NDCs and are younger at first recording. These findings likely reflect a combination of higher underlying need, differences in opportunities for identification and monitoring, and differences in the continuity of parental support and advocacy available to care-experienced children. Earlier diagnoses would facilitate more timely access to support, highlighting the potential benefits of multi-agency involvement among those with care experience.

## Introduction

There is growing awareness of the impact that neurodevelopmental conditions (NDCs) can have on the life trajectories of children and young people. These lifelong conditions can shape a child’s development, education, and emotional and behavioural functioning. NDCs are increasingly recognised as important determinants of long-term well-being, particularly when diagnoses are delayed or when support needs go unmet [1, 2]. However, despite rising interest in the public health implications of NDCs, few studies have examined the needs of care-experienced children – those less than 18 years old who are in the care of a local authority.

The number of children and young people diagnosed with NDCs is increasing, both globally and in the United Kingdom [3–5]. This may reflect improved awareness, changes in diagnostic criteria, and greater efforts to identify and support neurodivergent children in educational and healthcare settings. The Welsh Government’s Review of Neurodevelopmental Services (2022) reported a significant mismatch between demand for assessments and capacity within health services. This has resulted in delayed diagnoses, which can hinder access to support and exacerbate disadvantage [6]. More recent Welsh Government priorities highlight that these challenges remain ongoing, with demand for children’s neurodevelopmental assessments continuing to increase, which highlights the continued need to reduce waiting times [7].

We use the term “neurodevelopmental conditions” throughout, and then refer to the specific medicalised names of each condition used in the databases in the methods and results sections. We acknowledge that the neurodivergent community have different language preferences and by referring to the neurodivergent conditions by their medicalised names we are not endorsing a medicalised or deficit-based understanding of neurodivergence. NDCs include conditions affecting cognitive, behavioural, and social development, including attention deficit hyperactivity disorder (ADHD), autism spectrum disorder (ASD), specific learning difficulties (e.g. dyslexia, dyscalculia) and motor disorders such as tic disorders.

We sought to examine the recording of NDCs in health and education records among a particularly vulnerable population, those with care experience. In Wales, children looked after by local authorities are those who have been accommodated by the local authority for more than 24 hours or are subject to a care order or placement. The rate of children looked after (also known as children in care) has continued to increase, with 114 per 10,000 (n = 7,072) children aged less than 18 years in 2025 [8]. These children are known to experience higher levels of health inequality, adverse childhood experiences, and poorer educational outcomes compared to their peers [9–13].

Within this population, children with disabilities, including NDCs such as ASD and ADHD, are overrepresented in the care system [14–19]. Furthermore, children in care with disabilities often face more complex health and social needs, requiring coordinated and timely support across several services [20]. Despite this increased level of need, care-experienced children frequently encounter barriers to accessing timely support. For example, they are more likely to experience difficulties accessing mental health services, including being less likely to receive mental health support following referral [21], potentially exacerbating existing inequalities, and contributing to poorer long-term outcomes.

The increased prevalence of NDCs among children with care experience likely reflects a complex interplay between genetic vulnerability and environmental and social risk. Several parental factors associated with child removal, including parental mental health difficulties and substance misuse, share genetic links with ASD, ADHD, and other mental health conditions [22], suggesting that inherited or shared genetic predisposition may contribute to neurodevelopmental vulnerability.

While further evidence is needed, environmental exposures may also influence developmental trajectories. ASD has been associated with prenatal medication exposure, socioeconomic status, education, low birth weight and air pollution [23], while ADHD has been linked to parental interaction quality, parental relationship status, maltreatment and parental incarceration [24]. Prenatal exposure to alcohol and illicit substances has also been associated with increased risk of ASD and ADHD [25]. As children entering care may be more likely to experience such adversities, these cumulative exposures may contribute to their increased risk of NDCs.

This risk may accumulate across the life course, with adverse experiences continuing beyond the prenatal and early childhood periods. Exposure to maltreatment and other adverse childhood experiences may further affect developmental trajectories and increase support needs, while care placement instability and disruptions to familiar relationships and routines may exacerbate anxiety, frustration, and behavioural stress [26, 27]. Thus, the relationship between NDCs and care experience is likely multifactorial, reflecting the cumulative effects of biological, environmental, and social exposures before, during, and after childhood care experiences.

Failure to recognise NDC needs in care-experienced children may create additional barriers to accessing appropriate support. Identification of needs is essential to ensuring health, education, and social services can respond effectively to children’s needs [28]. However, despite the high levels of NDCs among this population, little is known about the recording of NDCs among care-experienced children at a national level. Existing research is often limited to local authority case studies or relies on self-reported data that lacks diagnostic precision and may provide an incomplete picture of need.

Using Welsh population-level data, this study examined the proportion of children with NDCs, compared diagnostic proportions between children with and without care experience, and described the characteristics of children with NDC diagnoses according to care experience. It also explored age at diagnosis according to care experience and assessed whether diagnoses of NDCs differed by data source for children with and without care experience.

## Methods

### Study Design

Using a retrospective population-based cohort design, we investigated NDCs and care experience using data held within the Secure Anonymised Information Linkage (SAIL) Databank. SAIL is a data repository containing person-level de-identified health, education, social and criminal justice administrative data covering the population of Wales. Anonymised data can be accessed via a Trusted Research Environment, with access governed by an established Information Governance model. All data within the SAIL Databank are treated in accordance with the Data Protection Act 2018 and are compliant with the General Data Protection Regulation.

### Data Sources and Linkage

Within SAIL, person-level records can be linked between datasets, for research purposes, using the Anonymised Linkage Field (ALF). The linkage process has been described [28–30]. In brief, individuals are assigned an ALF by a third party based on person identifiers, such as their National Health Service (NHS) number, name, sex, date of birth and postcode.

The Welsh Demographic Service Dataset (WDSD) was used to extract demographic characteristics, including age, sex, address history and area-level deprivation. Area-level deprivation was measured using the Welsh Index of Multiple Deprivation (WIMD). We used the Annual District Death Extract to ascertain whether the child was alive at the start of each Key Stage (KS). A ‘Key Stage’ is a block of years within Wales’ education system that defines the national curriculum and expected educational knowledge of students at specific ages.

We obtained data on NDCs and their treatment (i.e. prescriptions relating to diagnoses) using primary care (Welsh Longitudinal General Practice Dataset) and secondary care datasets (Patient Episode Database for Wales [inpatient hospital admissions] and Outpatient Dataset). At the time of analysis, primary and secondary care data were available for 86% and 100% of the Welsh population, respectively.

The education dataset (Pupil Level Annual School Census, PLASC) provides information relating to special educational needs (SEN) provisioning and SEN type, as well as eligibility for free school meals (FSM). The PLASC is a collection of data provided to Welsh Government on all children attending a state-maintained school.

The national Children Looked After dataset was used to identify children with care experience within a given fiscal year (1 April to 31 March). This dataset contains information about the child’s care episode (placement type, legal status, category of need) and demographic data such as age, sex and ethnicity from 1 April 2002 to 31 March 2021 [31].

### Study Cohort

The available data consisted of all children born between 1^st^ September 2002 and 31^st^ August 2015. We examined the data for all school-aged children (5 – 16 years old) based on their age at the end of the academic year (31^st^ August, Appendix 1). Children born between 1^st^ September 2002 and 31^st^ August 2006 who had potential for follow-up to the end of Key Stage 4 (KS4; ages 14 – 16 years) formed the study cohort (Figure 1).

**Figure 1:**
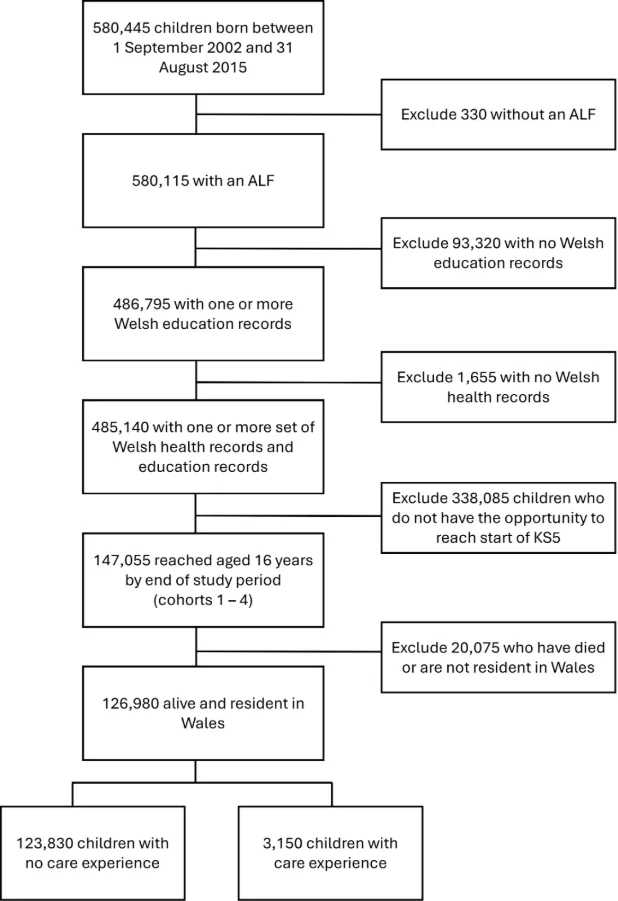
Consort Depicting the Cohort Production

We further restricted the cohort using several criteria. All children were required to have an ALF to enable linkage between education, health, and children’s social care datasets. In addition, children were required to be a resident in Wales for at least 11 months (>335 days) in the year prior to the academic KS of interest to ensure that the opportunity arose for a child to have an NDC recorded within their health records. The final sample consisted of 126,980 children.

### Measures

#### Care Experience Status

A child was considered to have care experience if they had a care episode recorded within the Children Looked After dataset during KS4 (age 14 – 16 years). Children with care experience are those in the care of the local authority or provided with accommodation for a continuous period of more than 24 hours. A binary flag (yes/no) was created. Children can be placed in respite care, to provide a temporary short-term break for families and caregivers of children with disabilities or special needs. We included these children in the analysis because many children entering care for respite reasons often do so because of disability-related support needs. Excluding them could therefore exclude an important subgroup of disabled children with care experience. However, recognising that this inclusion may influence comparisons between groups, we conducted a sensitivity analysis excluding children who were ever recorded as having respite care (see Statistical Analysis).

#### Neurodevelopmental Conditions Diagnosis

From here on, we refer to NDCs identified by SEN type, diagnosis, or prescription codes as diagnoses. The following NDCs were identified: ASD, learning difficulties, specific learning difficulties (ADHD, dyslexia, dyscalculia, and dyspraxia) and tic disorder. The overall counts across all NDCs were summarised as NDC.

To identify NDCs within the healthcare data, we used clinical code lists developed in previous studies (Appendix 2) [32–35]. We used Read codes (version 2) used within general practices (GP) [36], and International Classification of Diseases version 10 (ICD-10) codes used in secondary care, to attain information on diagnoses and prescriptions. Within the education data, we categorised those with moderate learning difficulties, severe learning difficulties, profound and multiple learning difficulties, and speech, language and communication difficulties as having learning difficulties. In 2011/2012 changes to the recording practices in SEN types captured in the education data resulted in specific learning difficulties being separated into their own SEN type. This resulted in separate codes for ADHD, dyslexia, dyscalculia, and dyspraxia, which were previously combined.

ASD, learning difficulties and ADHD diagnoses could be identified in both the health and education data, while some specific learning difficulties including dyslexia, dyspraxia, and dyscalculia were only available within the education data (excluding ADHD). Tic disorders could only be identified through the healthcare datasets. The source (health or education) of the recorded NDC was compared between children with and without care experience for NDCs identifiable through both health and education datasets (ASD, learning difficulties and ADHD). Specific learning difficulties (dyslexia, dyscalculia, and dyspraxia) were excluded from this analysis because they were only identifiable through education records. Similarly, tic disorders were not examined because they are only identifiable through healthcare records.

#### Children’s Characteristics

We calculated the age of the child at the time of their healthcare diagnosis. For diagnoses identified through education records (PLASC Census), the child’s age at the start of the academic year in which the diagnosis was recorded was used as the exact date of diagnosis was not available. Although the PLASC Census is always collected in January each year, the exact date changes annually. We also captured whether the child was eligible for FSM, SEN provisioning (no SEN and SEN), and the school setting they were enrolled in by the start of KS5 (maintained, special or education otherwise than at schools (EOTAS)). This part of the analysis resulted in a slightly different cohort, as although children remained residents in Wales during KS4, not all children were enrolled in a school for KS4.

Deprivation scores were derived using the WIMD 2019 quintiles according to Lower Super Output Area (LSOA) of residence. The WIMD uses quintiles to categorise small areas (LSOAs) based on their relative level of deprivation, where quintile 1 represents the most deprived and quintile 5 represents the least deprived [37]. The WIMD was assessed at the end (31 August) of KS4.

### Statistical Analysis

Data were prepared and extracted using Structured Query Language (SQL) via Eclipse and analysed using SPSS Statistics and R Studio (version 4.1.3). We summarised the cohort using descriptive statistics and calculated proportions (per 10,000) of diagnoses for children with and without care experience, including children with short breaks (respite care), and children without care experience. We repeated comparisons of the proportions of NDCs after excluding children who were ever recorded as having respite care. This analysis examined whether the inclusion of children receiving respite care influenced differences in recorded proportions of NDCs. We compared age at first recorded diagnosis between children with and without care experience. The distribution of age at diagnosis was assessed using Q-Q plots, which showed that the data were skewed. Therefore, we used the Mann-Whitney U test to determine median age at diagnosis and interquartile ranges (IQRs). The results are presented with the Mann-Whitney U test statistic (W) and p-values.

Children’s characteristics, including sex, FSM eligibility, SEN provisioning, and school type (mainstream vs other) were compared between those with and without care experience for each NDC using Pearson’s chi-square test of independence. Source of recorded diagnosis (health or education) was compared between those with and without care experience using Pearson’s chi-square test of independence.

In accordance with Higher Education Statistics Agency (HESA) compliance and requirements for disclosure control, counts were rounded to the nearest 5, and percentages were presented to zero decimal places. Proportions are calculated using unrounded counts, unless specified otherwise; where this was necessary to avoid disclosure risks.

## Results

### Cohort Characteristics by Care Experience

Of the 126,980 children (males, 51%), 3,150 (2%) were recorded as being ever looked after (Table 1). Of the children with care experience, 195 (6%) had ‘ever’ been recorded as having a short-term (respite) care episode. Overall, among the group with no care experience, there was a relatively even distribution of children across deprivation quintiles, with 23% in the most deprived and 20% in the least deprived areas. In contrast, children with care experience were disproportionately represented in the most deprived areas (32%) compared with the least deprived areas (11%).

**Table 1:** Percentage (and number) of Sex and Deprivation by Those With and Without Care Experience

	**No care experience % (n)**	**Care experience % (n)**
**Sex**		
Boys	51 (63,650)	52 (1,650)
Girls	49 (60,175)	48 (1,500)
**Missing**	(<10)	
**WIMD**		
1 (most deprived)	23 (28,080)	32 (1,015)
2	20 (25,130)	23 (740)
3	19 (23,120)	18 (550)
4	18 (22,850)	15 (480)
5 (least deprived)	20 (24,530)	11 (350)
Missing	<1 (120)	<1 (15)
**Total**	123,830	3,150

IMD: Welsh Index of Multiple Deprivation.

### Comparison of NDC proportions between those with and without care-experience

Among all children 37,525 (30%) had an NDC diagnosis (Table 2). Proportions of NDCs were higher among children with care experience compared to those without, at the end of Key Stage 4, particularly for learning difficulties and ADHD (Table 2). This pattern did not hold for all the specific learning difficulties. Proportions of dyslexia and dyspraxia were higher among those without care experience, while dyscalculia was more common among those with care experience. Tic disorder also had higher proportions among those with no care experience (61 vs 57 per 10,000). Overall, proportions of NDC were substantially higher among the care-experienced population (5,606 vs 2,888 per 10,000).

**Table 2:** Number and Proportions of NDCs Diagnoses at the End of Key Stage 4 Among Those With and Without Care Experience

	**No care experience**			**Care experience**		**Prevalence ratio**
	**(N=123,830)**			**(N=3,150)**		**(95% confidence intervals)**
	**Diagnosed**	**Proportions/10,000**		**Diagnosed**	**Proportions/10,000**	
ASD	3,880	313		275	876	2.79 (2.49–3.14)
Learning Difficulties	27,075	2,187		1,475	4,686	2.14 (2.06–2.23)
Specific Learning Difficulties	11,950	965		600	1,902	1.97 (1.83–2.12)
ADHD	3,230	261		365	1,152	4.42 (3.99–4.89)
Dyslexia	6,285	508		140	444	0.86 (0.74–1.03)
Dyspraxia	720	58		15	41	0.71 (0.41–1.22)
Dyscalculia	720	58		20	63*	1.11 (0.70–1.71)
Tic Disorder	760	61		20	57	0.99 (0.59–1.49)
NDC overall	35,760	2,888		1,765	5,606	1.94 (1.88–2.00)

Note. ^*^Proportions are based on rounded counts to prevent back-calculation of underlying counts.

In a sensitivity analysis, excluding children who had ever been in care for respite, proportions of NDC remained higher among children with care experience than those without care experience (Appendix 3). The pattern of findings across individual NDCs remained unchanged, indicating that the higher recorded proportions among children with care experience were not entirely attributed to the inclusion of children receiving respite care.

### Characteristics of Individuals with an NDC Diagnosis

Eligibility for FSM was significantly higher among children with care experience across diagnoses (p < 0.001); just under half of those with care experience were eligible compared to around a quarter of those with no care experience (Table 3). Children with care experience were also significantly more likely to have SEN provisioning across all diagnoses (*χ*^2^ = 21.3, p = 0.002), although proportions of provisioning were relatively similar for children diagnosed with ASD. Children with care experience were significantly less likely to be educated in mainstream schools compared to children with no care experience and the difference was most pronounced for children with ASD (49% with care experience vs 83% without). Across both populations, children with ASD were less likely to attend mainstream schools compared to those with other diagnoses.

**Table 3:** Educational Characteristics of Children with an NDC Diagnosis by Care Experience

	**No care experience**					**Care experience**					***χ* ^2^**		
	**Total**	**FSM eligibility**	**SEN provisioning**	**Mainstream school**		**Total**	**FSM eligibility**	**SEN provisioning**	**Mainstream school**		**FSM**	**SEN**	**Mainstream school**
		**(%, n)**	**(%, n)**	**(n, %)**			**(%, n)**	**(%, n)**	**school**				
ASD	265	30 (1,090)	94 (3,420)	83 (3,065)		3,675	44 (115)	98 (260)	49 (130)		21.33^**^	943*	177.87^**^
Learning difficulties	1,440	54 (14,035)	46 (12,135)	95 (24,975)		26,165	43 (615)	79 (1,130)	81 (1,160)		144.77^**^	339.23^**^	659.95^**^
Specific learning difficulties	560	24 (2,755)	72 (8,230)	96 (10,990)		11,485	42 (240)	89 (495)	76 (430)		100.76^**^	73.83^**^	432.27^**^
NDCs overall	1,705	26 (9,095)	57 (19,615)	96 (32,965)		34,445	43 (730)	79 (1,365)	81 (1,385)		220.24^**^	349.42^**^	815.47^**^

p-value significant at p < 0.01* < 0.001^**^.

Note: The number of children with a recorded NDC differs from that reported in Table 2, Table 4 and Table 5, as the cohort for this analysis was restricted to children who were enrolled within the education dataset by the start of KS5. Tic disorders were too small to be reported but were included in NDCs overall.

p-value significant at p <0.01* <0.001^**^.

Note: The number of children with a recorded NDC differs from that reported in Table 2, 4 and 5, as the cohort for this analysis was restricted to children who were enrolled within the education dataset by the start of KS5. Tic disorders were too small to be reported but were included in NDCs overall.

**Table 4:** Median Age at First Recorded NDC Diagnosis and Interquartile range (IQR) Among Those With and Without Care Experience

	**No care experience**			**Care experience**		**Mann-Whitney U**	**p-value**
	**Median age**	**IQR**		**Median age**	**IQR**	**statistic (W)**	
	**at diagnosis**			**at diagnosis**			
ASD	10.3	6.6–12.8		7.4	4.0–11.1	679,974	<0.0001
Learning difficulties	6.8	5.8–8.3		6.3	5.0–7.8	23,590,746	<0.0001
Specific learning difficulties	9.0	7.2–11.3		8.3	6.6–11.1	3,830,403	0.0037
NDCs overall	7.1	5.9–9.0		6.3	4.9–7.9	38,701,034	<0.0001

**Table 5:** Source of First Recorded NDC by Care Experience

	**No care experience**			**Care experience**		
	**Health (%, n)**	**Education (%, n)**		**Health (%, n)**	**Education (%, n)**	***χ* ^2^**
ASD	52 (2,035)	48 (1,845)		66 (180)	34 (95)	16.93^**^
ADHD	83 (2,680)	17 (550)		93 (335)	7 (25)	23.74^**^
Learning difficulties	3 (920)	97 (26,155)		7 (105)	93 (1,370)	54.88^**^

p-value <0.001^**^.

### Age at First Recorded NDC Diagnosis by Care Experience

Among children with a recorded NDC diagnosis, median age at diagnosis differed between children with and without care experience across several NDCs (Table 4). For ASD, the median age at diagnosis was significantly younger among those with care experience (7.4 years, 95% CI [6.3–8.5]) compared to those without care experience (10.3 years, 95% CI [10.1–10.5]). A similar pattern was observed for learning difficulties. For specific learning difficulties, median age at diagnosis was comparable between the two populations: 8.5 years among those with care experience compared to 9.0 years for those without care experience. When considering NDCs overall, children with care experience were diagnosed significantly younger than children with no care experience (6.3 years, 95% CI [6.2–6.4] vs 7.1 years, 95% CI [7.0–7.1]).

### Source of Recorded NDC

The source of recorded first NDC diagnosis differed between children with and with no care experience for ASD, ADHD and learning difficulties, although the trends followed similar directions (Table 4). Among children with no care experience, a diagnosis of ASD was similarly distributed between health and education data (52 vs 48%, respectively), whereas a greater proportion of ASD diagnoses among children with care experience were identified in the health data (66% vs 34%).

The majority of ADHD diagnoses were first recorded in health data for both groups, although the proportion recorded in health data was higher among children with care experience. In contrast, learning difficulties were predominantly recorded through education data in both groups, with only a small proportion identified through health records among children with no care experience.

## Discussion

### Summary of Main Findings

Proportions of NDCs among children with care experience were higher compared to those without care experience; this was particularly pronounced for learning difficulties and ADHD. Eligibility for FSM among children with NDCs was almost twice as high in care-experienced children. Children with care experience with an NDC were more likely to have SEN provisioning and were less likely to be in a mainstream school. Regarding age at first recorded diagnosis, children with care experience had a younger median age than children with no care experience, except for those with specific learning difficulties where age at diagnosis was comparable between groups.

Children with care experience and a specific learning difficulty, or ASD, were more likely to have a recorded diagnosis in the health data than the education data.

### Comparison with Previous Studies

This research builds on previous work showing that children receiving care, and those placed in care have high rates of disability [14]. We demonstrated that a range of NDCs are frequently recorded among children with care experience and extended previous research by using linked health and education records to identify NDC. Ogundele (2020) found that children in one English local authority were more likely to be neurodivergent or have emotional, behavioural or intellectual difficulties when compared to their peers with no care experience [38]. Parsons et al., (2019) found higher rates of autism diagnosis in one local authority for children in care compared to those not in care [39]. These findings are consistent with evidence from a nationwide Finnish birth cohort, which found higher proportions of NDCs, and particularly ADHD, among children with care experience compared to those who have never been in care [19, 40]. Despite differences in child welfare systems, the consistency of findings suggests that the association between ADHD and care experience is robust and unlikely to be explained solely by service use.

Differences in recording across health and education systems may reflect variation in assessment pathways and opportunities for identification. For example, ASD diagnoses were comparable in the source of recording for those with no care experience but higher proportions of health diagnoses among the care-experienced. For ADHD diagnoses, higher proportions were recorded in health data among both groups, whereas learning difficulties were predominantly identified through education records in both groups. These differences may reflect variation in how conditions are recognised, assessed and recorded across services, as well as differences in opportunities for contact with health care systems. For children with care experience, annual statutory health assessments and increased interactions with health and social care services may provide additional opportunities for early screening, identification and referrals. For example, ASD traits often emerge prior to school age, as early as one year old, meaning that they may be more likely to be detected during early years [41]. In contrast, learning difficulties may be more readily identified within education settings where children’s progress and needs are routinely monitored [42, 43].

However, differences in source of recorded NDCs should be interpreted with caution, as they may reflect differences in diagnostic pathways, data availability and underlying levels of need rather than differences in service provision alone. Earlier recorded diagnoses among children with care experience may also reflect differences in the complexity of developmental needs among children entering care, including those entering for respite care, as well as increased opportunities for identification through contact with multi-agency services.

### Strengths and Limitations

A key strength of this study is the use of population-level, linked administrative data, which enabled the inclusion of all children with NDCs born in Wales between 2002 and 2006 and followed from birth to age 16. The use of linked administrative data enabled inclusion of a large, population-level cohort, with findings generalisable to broader real-world populations and healthcare settings. We used multiple linked data sources, including health and education records, allowing robust identification of NDCs through educational records and clinical diagnostic and prescription records. The longitudinal design, following several birth cohorts from birth through to the end of Key Stage 4, enabled us to identify NDCs up until children were aged 16.

However, we acknowledge several limitations. The data were collected for administrative rather than research purposes, meaning that data quality may be variable and dependent on the completeness and accuracy of recording by local authorities and service providers. Education data on special educational needs (SEN) do not include the precise date of SEN identification; as a result, the child’s age at the start of the academic year of diagnosis was used as a proxy for diagnoses identified through education records. This may have introduced some imprecision into estimates of age at diagnosis and may also have affected the source of first recorded diagnosis (health versus education), as health records are updated continuously throughout the year, whereas education records are only captured in the annual PLASC Census (January). In addition, NDCs are increasingly being diagnosed among adults, which would not be captured in our analysis [4, 5]. The study also only captured children who had contact with public services or received formal diagnoses, meaning that children with unmet needs, private healthcare diagnoses, or undiagnosed NDCs were not included, likely resulting in an underestimate of true prevalence. It was not possible for us to capture information on the severity of NDCs within the available data, which limited exploration of how actual needs may relate to children’s social careinvolvement. Finally, we did not account for changes in care status during KS4. Therefore, we were unable to ascertain whether children were still in care or had returned home when their NDC was first recorded.

### Implications and Policy Relevance

While increased contact with health and social care services may provide additional opportunities for identification and support of NDCs among some care-experienced children, others may face barriers to timely identification of needs, due to disrupted education, frequent placement and school moves, and reduced continuity of parental support and advocacy [44]. Our findings are therefore likely to reflect a combination of higher needs and differences in opportunities for identification and recording. We consider these findings in the context of ongoing developments in neurodevelopmental pathways and services in Wales, which aim to improve recognition, assessment, and timely support for neurodivergent children [45].

Increased healthcare utilisation may indicate better recognition of need and proactive service engagement. This is consistent with wider developments of pathways and services in Wales, including the growing awareness of NDCs across agencies combined with the continued development of care and education pathways for children with NDCs e.g., Neurodivergence Improvement Programme and the all-Wales neurodevelopmental pathway [6, 45, 46]. Our findings highlight the need for children with care experience to be explicitly considered within these pathways, as their support needs may span across services and may require coordinated approaches across health, education, and social care systems.

These findings should also be considered in the context of substantial policy reforms in Wales, in particular the introduction of the Additional Learning Needs (ALN) and Educational Tribunal (Wales) Act [47]. Although implementation of this legislation post-dates the period covered by our analysis and therefore does not directly affect the findings presented here, it represents an important shift in how additional needs are identified and recorded. The transition from SEN to ALN marks a move from diagnosis-led to needs-led systems. Under this framework, all children identified with ALN are entitled to a statutory Individual Development Plan, and access to support is not reliant on a formal clinical diagnosis. Responsibility for identification of ALN now lies within educational settings, prior to receiving a healthcare [48]. While this approach has the potential to facilitate earlier support, it also introduces challenges for data comparability over time and across sectors. Further, while this fundamental shift may provide opportunities for earlier support for children without care experience, children with care experience may face additional challenges in accessing support because they are a highly mobile population, often experiencing school moves and educational disengagement [49]. Ensuring regular communication and coordination between services will therefore be important to ensure continuity of identification of children in care with NDCs and ongoing support.

The higher proportions of recorded NDCs among children with care experience reinforces their status as a high-risk group within policy and practice. Statutory health assessments, undertaken when children enter care and regularly throughout their time in care (six-monthly (0–5-year-olds) and annually (5– 18-year-olds)), provide opportunities to identify emerging health needs [50]. These regular health assessments may partly explain why children with care experience have younger age at first recorded NDC diagnosis. However, differences in age at recorded diagnosis may also reflect variation in underlying levels of need among children entering care.

Higher proportions of recorded NDCs among care-experienced children reinforce the need for coordinated support across health, education and social systems. Considerable variation in local provisioning, including access to specialist services (e.g., Children and Adolescent Mental Health Services) and support services and educational provision (e.g., Looked After Children Education (LACE) coordinators), may contribute to unequal access to timely assessment and support [51]. Our findings highlight the importance of ensuring that care-experienced children have access to assessments and support, regardless of the challenges experienced by local services.

Lastly, the findings have implications for educational provision. Higher proportions of SEN and lower enrolment in mainstream schools among care-experienced children suggest greater levels of need, although these patterns are also shaped by wider socioeconomic disadvantage [52]. As such, our findings strengthen the evidence base for targeted investment in schools with higher proportions of care-experienced children, as well as ensuring that mainstream educational settings are adequately resourced to meet the needs of children with NDCs. Without sufficient investment, there is a risk that needs will continue to outweigh the capacity of services to provide effective support.

In conclusion, children with care experience had higher proportions of NDCs than children with no care experience, highlighting the considerable neurodevelopmental needs within this population. Children with care experience were also diagnosed at a younger age. These findings are consistent with previous literature demonstrating that NDCs are overrepresented among care-experienced children, and may reflect a combination of higher underlying need, differences in opportunities for identification, and variation in recording across health and education services. Our findings highlight the importance of coordinated health, education, and social care pathways to support timely identification and access to services. The contributions of health and education datasets also demonstrate the value of linked administrative data for understanding the neurodevelopmental needs in this population.

## Supplementary Files

Supplementary Appendices

## Data Availability

This study makes use of anonymised, individual-level data held in the SAIL Databank, a Trusted Research Environment, at Swansea University, Swansea, UK. Due to the nature and level of the data, data are not publicly available. All proposals to use SAIL data are subject to review by the independent IGRP. The IGRP gives careful consideration to each project proposal to ensure proper and appropriate use of SAIL data. If a project is approved, access to the requested data is gained through a privacy-protecting safe haven and remote access system referred to as the SAIL Gateway. SAIL has established an application process to be followed by anyone who would like to access data via SAIL at: https://www.saildatabank.com/application-process/.
