# Supplementary material for: Neurodevelopmental Conditions Amongst Care-Experienced Children: A Population-Based Cohort Study Using Linked Health, Social Care, and Education Records from Wales
Source: Int J Popul Data Sci. 2026 Oct 6;11(1):3798. doi: 10.23889/ijpds.v11i1.3798 (PMC13679042; doi:10.23889/ijpds.v11i1.3798)
Supplement: Supplementary file 1 [file ijpds-11-3798-s001.pdf]

Supplementary Appendices

Appendix 1: The 13 cohorts and the data available for each cohort

WOB		Birthday during academic year																							
		2000/01	2001/02	2002/03	2003/04	2004/05	2005/06	2006/07	2007/08	2008/09	2009/10	2010/11	2011/12	2012/13	2013/14	2014/15	2015/16	2016/17	2017/18	2018/19	2019/20	2020/21	2021/22	2022/23	2023/24
01/09/2002	31/08/2003			0	1	2	3	4	5	6	7	8	9	10	11	12	13	14	15	16	17	18	19	20	21
01/09/2003	30/08/2004				0	1	2	3	4	5	6	7	8	9	10	11	12	13	14	15	16	17	18	19	20
01/09/2004	31/08/2005					0	1	2	3	4	5	6	7	8	9	10	11	12	13	14	15	16	17	18	19
01/09/2005	31/08/2006						0	1	2	3	4	5	6	7	8	9	10	11	12	13	14	15	16	17	18
01/09/2006	31/08/2007							0	1	2	3	4	5	6	7	8	9	10	11	12	13	14	15	16	17
01/09/2007	30/08/2008								0	1	2	3	4	5	6	7	8	9	10	11	12	13	14	15	16
01/09/2008	31/08/2009									0	1	2	3	4	5	6	7	8	9	10	11	12	13	14	15
01/09/2009	31/08/2010										0	1	2	3	4	5	6	7	8	9	10	11	12	13	14
01/09/2010	31/08/2011											0	1	2	3	4	5	6	7	8	9	10	11	12	13
01/09/2011	30/08/2012												0	1	2	3	4	5	6	7	8	9	10	11	12
01/09/2012	31/08/2013													0	1	2	3	4	5	6	7	8	9	10	11
01/09/2013	31/08/2014														0	1	2	3	4	5	6	7	8	9	10
01/09/2014	31/08/2015															0	1	2	3	4	5	6	7	8	9

Key: Green = pre-school, Yellow = statutory school age, Orange = optional education to age 18. We've only included the first four cohorts because they have complete data from KS1–KS4.

Appendix 2: Clinical codes used to identify neurodevelopmental conditions in health records

<table>
<tr><th colspan="2">Attention Deficit Hyperactive Disorder</th></tr>
<tr><th>Read Code v2</th><th>Description</th></tr>
<tr><td>6A61.</td><td>adhd annual review | attention deficit hyperactivity disorder annual review</td></tr>
<tr><td>8BPT.</td><td>drug therapy for adhd | drug therapy adhd (attention deficit hyperactivity disorder) | drug therapy for adhd (attention deficit hyperactivity disorder)</td></tr>
<tr><td>8BPT0</td><td>stimulant drug therapy adhd | stimulant drug therapy for adhd | stimulant drug therapy for adhd (attention deficit hyperactivity disorder)</td></tr>
<tr><td>8BPT1</td><td>non-stimulnt drug therapy adhd | non-stimulant drug therapy for adhd | non-stimulant drug therapy for adhd (attention deficit hyperactivity disorder)</td></tr>
<tr><td>9Ngp.</td><td>on drug therapy adhd | on drug ther adhd (attention deficit hyperactivity disorder) | on drug therapy for adhd (attention deficit hyperactivity disorder)</td></tr>
<tr><td>9Ngp0</td><td>on stimulant drug therapy adhd | on stim drug ther adhd (attention def hyperactivity disordr) | on stimulant drug therapy for adhd (attention deficit hyperactivity disorder)</td></tr>
<tr><td>9Ngp1</td><td>on non-stimulnt drug ther adhd | on non-stimulant drug therapy for adhd | on non-stimulant drug therapy for adhd (attention deficit hyperactivity disorder)</td></tr>
<tr><td>9Ol8.</td><td>adhd monitr invitatn 1st lettr | adhd monitoring invitation first letter | attention deficit hyperactivity disorder monitoring invitation first letter</td></tr>
<tr><td>9Ol9.</td><td>adhd monitr invitatn 2nd lettr | adhd monitoring invitation second letter | attention deficit hyperactivity disorder monitoring invitation second letter</td></tr>
<tr><td>9OlA.</td><td>adhd monitr invitatn 3rd lettr | adhd monitoring invitation third letter | attention deficit hyperactivity disorder monitoring invitation third letter</td></tr>
<tr><td>dw4..</td><td>Guanfacine</td></tr>
<tr><td>dw41.</td><td>INTUNIV 1mg m/r tablets</td></tr>
<tr><td>dw42.</td><td>GUANFACINE 1mg m/r tablets</td></tr>
<tr><td>dw43.</td><td>INTUNIV 2mg m/r tablets</td></tr>
<tr><td>dw44.</td><td>GUANFACINE 2mg m/r tablets</td></tr>
<tr><td>dw45.</td><td>INTUNIV 3mg m/r tablets</td></tr>
<tr><td>dw46.</td><td>GUANFACINE 3mg m/r tablets</td></tr>
<tr><td>dw47.</td><td>INTUNIV 4mg m/r tablets</td></tr>
<tr><td>dw48.</td><td>GUANFACINE 4mg m/r tablets</td></tr>
<tr><td>E2E..</td><td>childhood hyperkinetic syndr. | childhood hyperkinetic syndrome</td></tr>
<tr><td>E2E0.</td><td>child attention deficit disord | child attention deficit disorder</td></tr>
<tr><td>E2E00</td><td>attention deficit-not hyperact | attention deficit without hyperactivity</td></tr>
<tr><td>E2E01</td><td>attention deficit +hyperactive | attention deficit with hyperactivity</td></tr>
<tr><td>E2E0z</td><td>child attent.deficit dis.nos | child attention deficit disorder nos</td></tr>
<tr><td>E2E1.</td><td>hyperkinesis+development delay | hyperkinesis with developmental delay</td></tr>
<tr><td>E2E2.</td><td>hyperkinetic conduct disorder</td></tr>
<tr><td>E2Ey.</td><td>other hyperkinetic manifestat. | other hyperkinetic manifestation</td></tr>
<tr><td>E2Ez.</td><td>hyperkinetic syndrome nos</td></tr>
<tr><td>Eu90.</td><td>[x]hyperkinetic disorders</td></tr>
<tr><td>Eu900</td><td>[x]disturbance activity/attntn | [x]disturbance of activity and attention</td></tr>
<tr><td>Eu901</td><td>[x]hyperkinetic conduct disord | [x]hyperkinetic conduct disorder</td></tr>
<tr><td>Eu902</td><td>[x]def atten motor cont percep | [x]deficits in attention, motor control and perception</td></tr>
<tr><td>Eu90y</td><td>[x]oth hyperkinetic disorders | [x]other hyperkinetic disorders</td></tr>
<tr><td>Eu90z</td><td>[x]hyperkinetic disorder, unsp | [x]hyperkinetic disorder, unspecified</td></tr>
<tr><td>Eu9y7</td><td>[x]attention deficit disorder</td></tr>
<tr><td>ZS91.</td><td>attention deficil disorder</td></tr>
<tr><td>dc1..</td><td>dexamfetamine sulphate</td></tr>
<tr><td>dc11.</td><td>*dexedrine 5mg tablets</td></tr>
<tr><td>dc12.</td><td>*durophet 7.5mg m/r capsules</td></tr>
<tr><td>dc13.</td><td>*durophet 12.5mg m/r capsules</td></tr>
<tr><td>dc14.</td><td>*durophet 20mg m/r capsules</td></tr>
<tr><td>dc1w.</td><td>dexamfetamine sulph 5mg tabs | dexamfetamine sulphate 5mg tablets</td></tr>
<tr><td>dc1x.</td><td>*dexamphetamine 7.5mg m/r caps | dexamphetamine sulphate 7.5mg m/r capsules</td></tr>
<tr><td>dc1y.</td><td>*dexamphetamine 12.5mg caps | dexamphetamine sulphate 12.5mg m/r capsules</td></tr>
<tr><td>dc1z.</td><td>*dexamphetamine 20mg m/r caps | dexamphetamine sulphate 20mg m/r capsules</td></tr>
<tr><td>dw1..</td><td>methylphenidate</td></tr>
<tr><td>dw11.</td><td>methylphenidate hcl 10mg tabs | methylphenidate hydrochloride 10mg tablets</td></tr>
</table>

Continued

Appendix 2: Continued

Attention Deficit Hyperactive Disorder	
**Read Code v2**	**Description**
dw12.	ritalin 10mg tablets
dw13.	*equasym 5mg tablets
dw14.	*equasym 20mg tablets
dw15.	*equasym 10mg tablets
dw16.	equasym xl 20mg m/r capsules
dw17.	concerta xl 18mg m/r tablets
dw18.	concerta xl 36mg m/r tablets
dw19.	*tranquilyn 5mg tablets
dw1A.	*tranquilyn 10mg tablets
dw1B.	*tranquilyn 20mg tablets
dw1C.	equasym xl 10mg m/r capsules
dw1D.	equasym xl 30mg m/r capsules
dw1E.	medikinet xl 10mg m/r capsules
dw1F.	medikinet xl 20mg m/r capsules
dw1G.	medikinet xl 30mg m/r capsules
dw1H.	medikinet xl 40mg m/r capsules
dw1I.	concerta xl 27mg m/r tablets
dw1J.	medikinet 5mg tablets
dw1K.	medikinet 10mg tablets
dw1L.	medikinet 20mg tablets
dw1M.	medikinet xl 5mg m/r capsules
dw1q.	methylphenidate 5mg m/r caps | methylphenidate hydrochloride 5mg m/r capsules
dw1r.	methylphenidate 27mg m/r tabs | methylphenidate hydrochloride 27mg m/r tablets
dw1s.	methylphenidate 40mg m/r caps | methylphenidate hydrochloride 40mg m/r capsules
dw1t.	methylphenidate 10mg m/r caps | methylphenidate hydrochloride 10mg m/r capsules
dw1u.	methylphenidate 30mg m/r caps | methylphenidate hydrochloride 30mg m/r capsules
dw1v.	methylphenidate 36mg m/r tabs | methylphenidate hydrochloride 36mg m/r tablets
dw1w.	methylphenidate 18mg m/r tabs | methylphenidate hydrochloride 18mg m/r tablets
dw1x.	methylphenidate 20mg m/r caps | methylphenidate hydrochloride 20mg m/r capsules
dw1y.	methylphenidate hcl 5mg tabs | methylphenidate hydrochloride 5mg tablets
dw1z.	methylphenidate hcl 20mg tabs | methylphenidate hydrochloride 20mg tablets
dw2..	atomoxetine
dw21.	strattera 10mg capsules
dw22.	strattera 18mg capsules
dw23.	strattera 25mg capsules
dw24.	strattera 40mg capsules
dw25.	strattera 60mg capsules
dw26.	strattera 80mg capsules
dw2u.	atomoxetine 80mg capsules
dw2v.	atomoxetine 60mg capsules
dw2w.	atomoxetine 40mg capsules
dw2x.	atomoxetine 25mg capsules
dw2y.	atomoxetine 18mg capsules
dw2z.	atomoxetine 10mg capsules
dw3..	lisdexamfetamine
dw31.	elvanse 30mg capsules
dw32.	elvanse 50mg capsules
dw33.	elvanse 70mg capsules
dw3x.	lisdexamfetamine dim 70mg caps | lisdexamfetamine dimesylate 70mg capsules
dw3y.	lisdexamfetamine dim 50mg caps | lisdexamfetamine dimesylate 50mg capsules
dw3z.	lisdexamfetamine dim 30mg caps | lisdexamfetamine dimesylate 30mg capsules

Continued

Appendix 2: Continued

**ICD code**	**Description**
**F90**	**hyperkinetic disorders**
F90.0	disturbance of activity and attention
F90.1	hyperkinetic conduct disorder
F90.2	attention deficit hyperactivity disorder, combined type
F90.8	other hyperkinetic disorders
F90.9	hyperkinetic disorder, unspecified
	**Autism Spectrum Disorder**
**Read Code v2**	**Description**
E140.	infantile autism
E1400	infantile autism – active | active infantile autism
E1401	infantile autism – residual | residual infantile autism
E140z	infantile autism nos
Eu840	[x]childhood autism
Eu841	[x]atypical autism
Eu845	[x]asperger's syndrome
1J9..	suspected autism
**ICD code**	**Description**
F84.0	Childhood autism
F84.1	Atypical autism
F84.2	Rett's syndrome
F84.3	Other childhood disintegrative disorder
F84.4	Overactive disorder associated with mental retardation and stereotyped movements
F84.5	Asperger's syndrome
F84.9	Pervasive developmental disorders, unspecified
	**Learning Difficulties**
**Read Code v2**	**Description**
R0463	[d]dyslexia
2B55.	o/e - dyslexia
E2F02	developmental dyslexia
Eu810	[x]specific reading disorder
Eu81.	[x]specif scholastic devel dis | [x]specific developmental disorders of scholastic skills
Eu81z	[x]dev dis scholas skills unsp | [x]developmental disorder of scholastic skills, unspecified
Eu80z	[x]dev disord speech/lang unsp | [x]developmental disorder of speech and language unspecified | [x]developmental disorder of speech and language, unspecified
Eu800	[x]specif speech articulat dis | [x]specific speech articulation disorder
2B5..	o/e - symbolic dysfunction
R046.	[d]other symbolic dysfunction
Eu83.	[x]mix specific develop disord | [x]mixed specific developmental disorders
Eu811	[x]specific spelling disorder
Eu81y	[x]oth dis scholastic skills | [x]other developmental disorders of scholastic skills
Eu801	[x]expressive language disordr | [x]expressive language disorder
8E23.	dyslexia training
Eu82.	[x]spec devel disor motor func | [x]specific developmental disorder of motor function
Eu813	[x]mixed scholastic skill dis | [x]mixed disorder of scholastic skills
Eu80.	[x]spec develop dis speech/lan | [x]specific developmental disorders of speech and language
Eu812	[x]specific disord arithmetic | [x]specific disorder of arithmetical skills
**ICD code**	**Description**
F81	specific developmental disorders of scholastic skills
F81.0	specific reading disorder
F81.8	other developmental disorders of scholastic skills
F81.9	developmental disorder of scholastic skills, unspecified
F80.9	developmental disorder of speech and language, unspecified

Continued

Appendix 2: Continued

Learning Difficulties	
**Read Code v2**	**Description**
F80.0	specific speech articulation disorder
F83	mixed specific developmental disorders
F81.1	specific spelling disorder
F80.1	expressive language disorder
F82	specific developmental disorder of motor function
F81.3	mixed disorder of scholastic skills
F80	specific developmental disorders of speech an language
F81.2	specific disorder of arithmetical skills
R48	dyslexia and other symbolic dysfunctions, not elsewhere specified
R48.0	dyslexia and alexia

Tic Disorder	
**Read Code v2**	**Description**
2974	o/e - spasm/tic
2974.12	o/e - tic
1B24.00	has a tic
1B24.11	tic - symptom
E272.00	tics
E272000	tic disorder unspecified
E272100	transient childhood tic
E272200	chronic motor tic disorder
E272300	gilles de la tourette's disorder
E272z00	tic nos
Eu95.00	[x]tic disorders
Eu95000	[x]transient tic disorder
Eu95100	[x]chronic motor or vocal tic disorder
Eu95200	[x]comb vocal multiple motor tic disorder - de la tourette
Eu95y00	[x]other tic disorders
Eu95z00	[x]tic disorder, unspecified
F133.00	tics of organic origin
**ICD code**	**Description**
F95	Tic Disorder
F95.0	Transient tic disorder
F95.1	Chronic motor or vocal tic disorder
F95.2	De la Tourette
F95.8	Other tic disorders
F95.9	Tic disorder, unspecified

Appendix 3: Sensitivity analysis of recorded NDC proportions after excluding children receiving respite (short breaks) care

	**No care experience (N=123,830)**		**Care experience (N=3,150)**		**Care experience (excluding respite) (N=2,955)**	
	**Diagnosed**	**Proportions/ 10,000**	**Diagnosed**	**Proportions/ 10,000**	**Diagnosed**	**Proportions/ 10,000**
ASD	3,880	313	275	876	175	599
Learning Difficulties	27,075	2,187	1,475	4,686	1,320	4,464
Specific Learning Difficulties	11,950	965	600	1,902	535	1,813
ADHD	3,230	261	365	1,152	310	1,042
Dyslexia	6,285	508	140	444	140	467
Dyspraxia	720	58	15	41	<>	<>
Dyscalculia	720	58	20	63*	20	68*
Tic Disorder	760	61	20	57	<>	<>
NDC overall	35,760	2,888	1,765	5,606	1,585	5,364

Note. Tic Disorder and Dyspraxia were masked (denoted as <>), in line with disclosure control, due to disclosure risk (differences <10); *Proportions are based on rounded counts to prevent back-calculation of underlying counts.
